# Global transmission of monkeypox virus—a potential threat under the COVID-19 pandemic

**DOI:** 10.3389/fimmu.2023.1174223

**Published:** 2023-05-05

**Authors:** Yang Wang, Ping Leng, Hao Zhou

**Affiliations:** College of Medical Technology, Chengdu University of Traditional Chinese Medicine, Chengdu, China

**Keywords:** monkeypox virus, transmission, airline travel, detection, vaccination

## Abstract

Monkeypox virus (MPXV) cases have increased dramatically worldwide since May 2022. The Atlanta Center for Disease Control and Prevention (Atlanta CDC) had reported a total of 85,922 cases as of February 20^th^, 2023. During the COVID-19 pandemic, MPXV has emerged as a potential public threat. MPXV transmission and prevalence must be closely monitored. In this comprehensive review, we explained the basic characteristics and transmission routes of MPXV, individuals susceptible to it, as well as highlight the impact of the behavior of men who have sex with men (MSM) and airline traveling on recent outbreaks of MPXV. We also describe the clinical implications, the prevention of MPXV, and clinical measures of viral detection.

## 1 Introduction

While the world is still challenged by the COVID-19 pandemic, a global outbreak of the monkeypox virus (MPXV) poses a new potential threat to public health (1). A total of 85,922 cases had been reported as of February 20^th^, 2023 (Atlanta CDC). MPXV, a member of the Orthopoxvirus genus and Poxviridae family, encoding approximately 190 open reading frames, is a zoonotic double-stranded DNA virus with brick-shaped morphology (2–4). The core region of MPXV, which encodes essential enzymes and structural proteins, is 96.3% identical to that of vaccinia virus (VACV) (5). In this review, we discuss the characteristics and genetic evolution of MPXV and the epidemiological characteristics observed in this unprecedented outbreak, particularly the impacts of international air travel and the behavior of men who have sex with men (MSM). Clinically, understanding the syndromes of patients with MPXV infection and methods for preventing and detecting the virus would aid health workers in rapidly identifying the MPXV and improving public health.

## 2 Orthopoxvirus and MPXV classification

Besides MPXV, orthopoxvirus members that cause human diseases also include variola virus (VARV), cowpox virus (CPXV), and VACV. VARV is the causative agent of lethal smallpox disease (6). Contrasting to some of the other orthopoxviruses, the only known reservoir of VARV is humans (7). In the 20^th^ century, the rapid spread of VARV caused 300−500 million deaths (7). VACV, which has high sequence conservation with VARV, was used to eradicate smallpox infection in the past. It induces zoonotic diseases that are mainly associated with the handling of infected dairy cattle (8, 9). CPXV induces a self-limited disease and transmits to humans through direct contact with infected animals (mostly cats), albeit human CPXV infection occurs rarely (10, 11). The Central African (Congo Basin) clade and the West African clade are two distinct genetic clades of the MPXV (12). However, to use a non-discriminatory and non-stigmatizing nomenclature of MPXV, the two clades were later renamed as clade I (corresponds to the prior “Congo Basin clade”) and clade II (corresponds to the prior “West African clade”) (13). Clade I is considered more virulent, with a case fatality ratio (CFR) >10% whereas clade II is less fatal, with a CFR <1% (14). Clade I encodes the monkeypox inhibitor of complement enzyme (MOPICE) to evade host immune attack while clade II does not (15). Similarly, clade I encodes the B14R protein (CPXV BR-209 protein orthologs), which competes with IL-1 for binding to the interleukin-1β receptor (16). Clade II comprises two subclades—clade IIa and clade IIb, with the latter including the the variants circulating during the 2022 global outbreak (13). Fortunately, clade II appears to be less specialized in immune evasion compared to clade I.

## 3 The history of MPXV

MPXV was first discovered in 1958 in two cynomolgus monkeys shipped from Singapore to Copenhagen (17) (**Table 1**). Later, in the first year of the 1970s, six human cases infected with the MPXV were reported in three West African countries; Liberia, Sierra Leone, and Nigeria (18). In 1980, smallpox vaccinations were widely discontinued after its eradication (7). Given the high genetic similarity between MPXV and VARV, the cross-reactive antibodies produced by the smallpox vaccine could provide indirect protection against MPXV infection (5). With the cessation of smallpox vaccination, MPXV returned in the 1990s; 88 confirmed cases were reported in the Democratic Republic of Congo between 1996 to 1997 (19). From 1980 to 2000, the prevalence of MPXV infection was majorly limited within the African continent (22, 23). In 2003, the first MPXV outbreak outside of Africa was reported in the United States (US) where 47 confirmed and/or suspected cases were reported (20). After almost 2 decades, the United Kingdom (UK) reported an imported MPXV case in May 2022 (24). Since then, MPXV infections have begun to spread widely outside of the African continent. As of February 20^th^, 2023, the outbreak has caused over 85,000 confirmed cases and 90 deaths in areas where monkeypox (mpox) had not been previously reported (Atlanta CDC).

**Table 1:** Timeline of the key events of the monkeypox pandemic.

Time	Locations	Cases	References
1958	Copenhagen	2 cynomolgus monkeys	(17)
1970 - 1971	Liberia, Sierra Leone, and Nigeria	6 human cases	(18)
1996 - 1997	Democratic Republic of Congo	88 human cases	(19)
2003	United States of America	47 human cases	(20)
As of February 20^th^, 2023	Worldwide	85,922 human cases	(21)

## 4 Genetic evolution of MPXV

The phylogenetic analysis indicates that lineage B.1 of MPXV clade IIb is the causative subtype of the viral outbreak in 2022 (25). The genome of MPXV is approximately 197 kb in length (26). 46 single nucleotide polymorphisms (SNPs) were found in the B.1 MPXV sequence (25, 27). Three non-synonymous SNPs (D209N, P722S, and M1741I) of the immunogenic surface glycoprotein, B21R, may improve MPXV transmission and immune evasion (27). Moreover, the mutations of the virus are demonstrated to be the primary effect of the host immunity’s selective pressure (28). In-depth mutation analysis, for example, revealed that the host apolipoprotein B mRNA-editing catalytic polypeptide-like 3 (APOBEC3) enzyme may cause viral mutations in the accelerated evolution of MPXV according to the GA > AA and TC > TT mutational bias of SNPs (25, 29). Similarly, Gigante et al. demonstrated that host APOBEC3 editing is a recurrent and dominant cause of MPXV evolution (30). Additionally, APOBEC3 has been identified to preferentially mutate DNA structures formed by inverted repeats in the MPXV genome (31). It is currently unknown what effects these mutations may have. Understanding the genetic evolution of MPXV will be critical for studying and controlling the MPXV.

## 5 MPXV transmission

**Figure 1:**
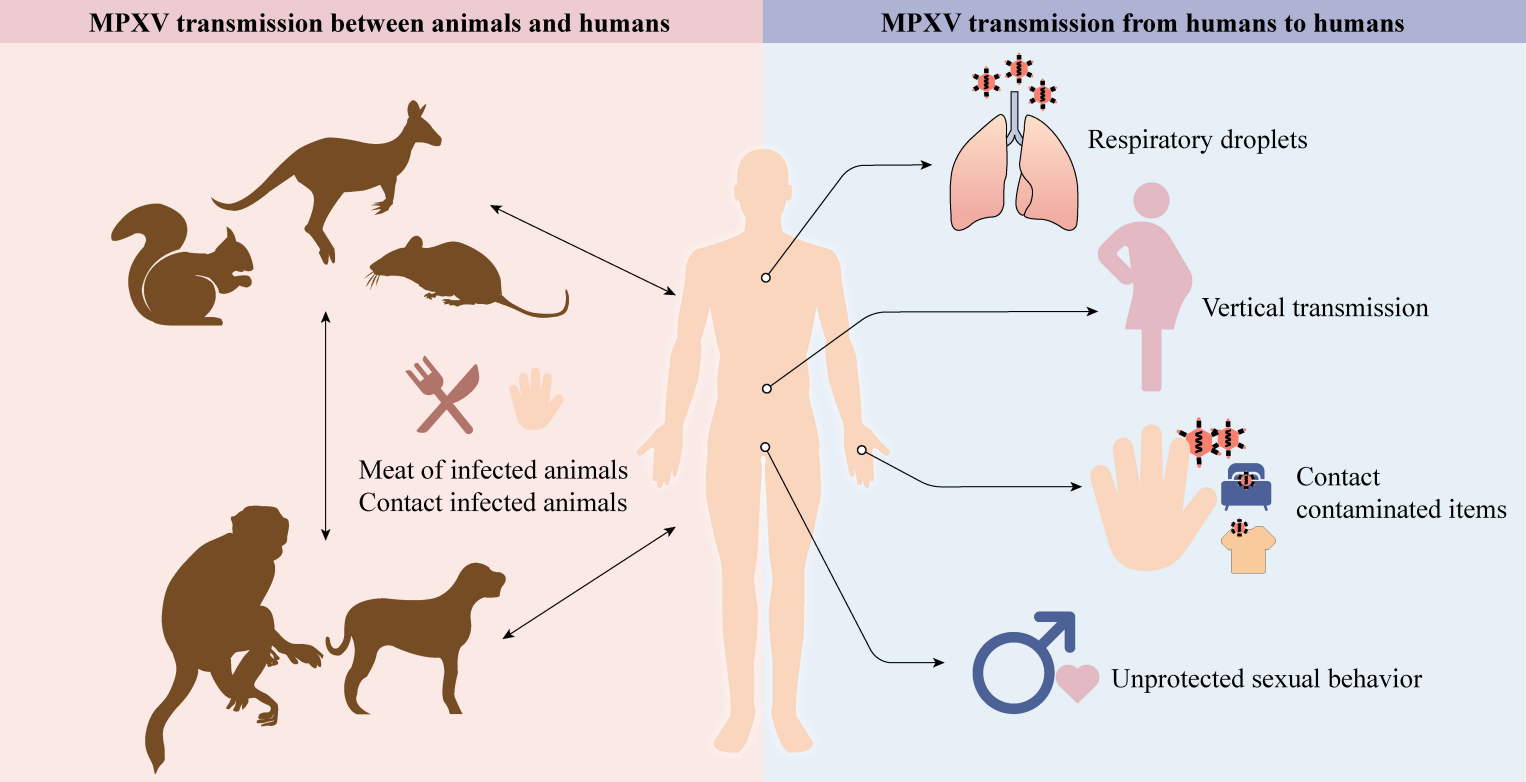
Animals, like rodents, dogs, and non-human primates, can spread MPXV to humans (cartoons modified from the SciDraw website). Respiratory droplets, vertical transmission, contact with contaminated items, and unprotected sexual behavior enable MPXV to transmit between humans.

MPXV can lead to severe zoonotic disease and be transmitted from animals to humans, humans to animals, and humans to humans (**Figure 1**).

### 5.1 MPXV transmission between animals and humans

In contrast with VARV, which has only one known human host, MPXV has a diverse host range, which may improve adaptation and transmission efficiency to humans. The natural reservoirs of MPXV include squirrels, Gambian pouched rats, dormice, and non-human primates (3, 32, 33). Exposure to excretions and secretions of infected pet prairie dogs caused an outbreak of 47 human cases in the US in 2003. Epidemiologic research has revealed that these dogs had a history of close contact with rodents imported from Ghana (20, 34). Similarly, the Central African Republic reported 5 confirmed cases originating from a patient who had come in contact with infected wild fauna (35). Surprisingly, human-to-pet transmission has also been documented (36). Although pet owners are at risk of transmitting their pets with MPXV, the risk remains low (37).

### 5.2 MPXV transmission from humans to humans

#### 5.2.1 Sexual transmission

Previously, the human-to-human transmission was believed to be involved in the outbreaks; however, the potential for sustained human-to-human transmission was believed to be low. Sexual transmission of MPXV is currently speculated to be a major contributor to transmission (**Table 2**). For example, Antinori et al. reported that 4 infected young adults in Italy were exposed to unprotected sexual encounters, and their seminal fluids tested positive for MPXV (38). Vivancos et al. also reported that 66 of 86 confirmed MPXV cases were reported by men who identified as gay, bisexual, or MSM (39). More significantly, Girometti et al. reported that all 54 cases at one health center in the UK were MSM (45). MSM accounts for a high percentage in each of these clinical cases. This is true in larger surveys with large sample sizes and respondents from multiple countries. A recent meta-analysis including 124 MPXV cases demonstrated that unprotected sexual behavior is the primary mode of transmission route (46). Similarly, 95% of infections in an investigation involving 528 MPXV infection cases across 16 countries from April to June 2022 are suspected to be transmitted through sexual intercourse (43). The CDC reported 1,195 patients until July 27, 2022, 99% of whom were men, and 94% of these men were reported to have had male-to-male sexual or intimate contact in the 3 weeks before symptoms began (44). However, it is important to note that these cases are not solid evidence of sexual transmission as MPXV can spread through non-sexual means, such as skin-to-skin contact or respiratory droplets when those individuals are close.

**Table 2:** Current confirmed cases of monkeypox with MSM between May 2022 and September 2022.

Locations	Confirmed cases	Confirmed cases with MSM (percentage)	Reported Time	References
Italy	4	4 (100%)	May 2022	(38)
UK	86	66 (77%)	From May 7^th^, 2022 to May 25^th^, 2022	(39)
Portugal	19	18 (95%)	As of May 27^th^, 2022	(40)
Spain	48	42 (88%)	From May 2022 to June 2022	(41)
UK	197	196 (99%)	From May 2022 to July 2022	(42)
Multi-regions	528	517 (98%)	From April 27^th^, 2022 to 24^th^, June 2022	(43)
US	1195	1123 (94%)	As of July 27^th^, 2022	(44)

#### 5.2.2 Non-sexual transmission

Non-sexual contact transmission mainly includes vertical transmission, respiratory droplets, skin-to-skin contact, and direct contact with contaminated items. Within the placenta, viral-resistant syncytiotrophoblast barriers can be overcome by orthopoxvirus entry mechanisms (47). Vertical transmission has been confirmed since stillbirths born to MPXV-infected pregnant women have a widespread rash, and MPXV DNA has been detected in fetal tissue, umbilical cord, and placenta (48). Large respiratory droplets containing aerosolized MPXV may cause human-to-human transmission during close and prolonged face-to-face contact (2). For example, a case of MPXV was reported in a traveler who had no recent sexual contact with infected people. His primary risk factor was non-sexual contact with numerous strangers at a crowded outdoor event (49). In a non-human primate experiment, cynomolgus monkeys became infected after being exposed to aerosolized MPXV and eventually died from pneumonia (50). This phenomenon was also observed in the black-tailed prairie dog model following intranasal administration of MPXV (51). Moreover, direct contact with an infected person’s rash, sores, scabs, or body fluids can spread MPXV (52). Inadvertent contact with contaminated items, such as clothing and bedding, can also spread MPXV (53–55). Therefore, the general public should be on the lookout for proper personal hygiene.

## 6 Susceptible individuals at high risk of exposure to MPXV

Newborns, pregnant women, children, and people with potential immune deficiencies are relatively more susceptible to MPXV infection, with a higher risk of severe complications and higher mortality rates. Newborns and pregnant women are at high risk of death and serious illness owing to their weakened immune systems (47). Mbala et al. reported that only one of the four pregnant women with MPXV had a healthy infant whereas the other two had miscarriages and one had fetal death, with stillbirths displaying diffuse skin lesions on the head, trunk, and extremities (48). However, the cause of fetal death remains elusive. Furthermore, children are vulnerable to MPXV. The Florida Department of Health (FDOH) reported a confirmed case of mpox in an infant younger than 2 months old in August 2022, making it the state’s youngest patient (56). Additionally, Huhn et al. demonstrated that pediatric patients (≤18 years old) have worse outcomes, such as being admitted to an intensive care unit (57). Furthermore, the CD4^+^ T cell counts in human immunodeficiency virus (HIV) infected individuals are typically low, making them vulnerable to severe diseases if exposed to MPXV (58). These patients with complex pathologies may provide a suitable environment for viruses to evolve and acquire mutations, making MPXV more virulent and transmissible (59). For example, the first person who died by MPXV outside of Africa was also infected by HIV (60). A retrospective review of 40 patients with MPXV hospitalized in Nigeria between September 2017 and December 2018 revealed that HIV-infected patients were likely to develop more severe lesions, were more susceptible to secondary bacterial skin infections, and had a longer disease duration (61). Additionally, HIV-positive patients account for a sizeable proportion of the reported MPXV cases. Among 27 confirmed cases of MPXV in Portugal, 14 cases (52%) were HIV-1-coinfected (40). Similarly, Bragazzi et al. reported that HIV-positive cases accounted for 54.29% of all 124 MPXV-confirmed cases in Italy, Australia, Portugal, the Czech Republic, and the UK (46).

## 7 The overview of the viral spread worldwide

**Figure 2:**
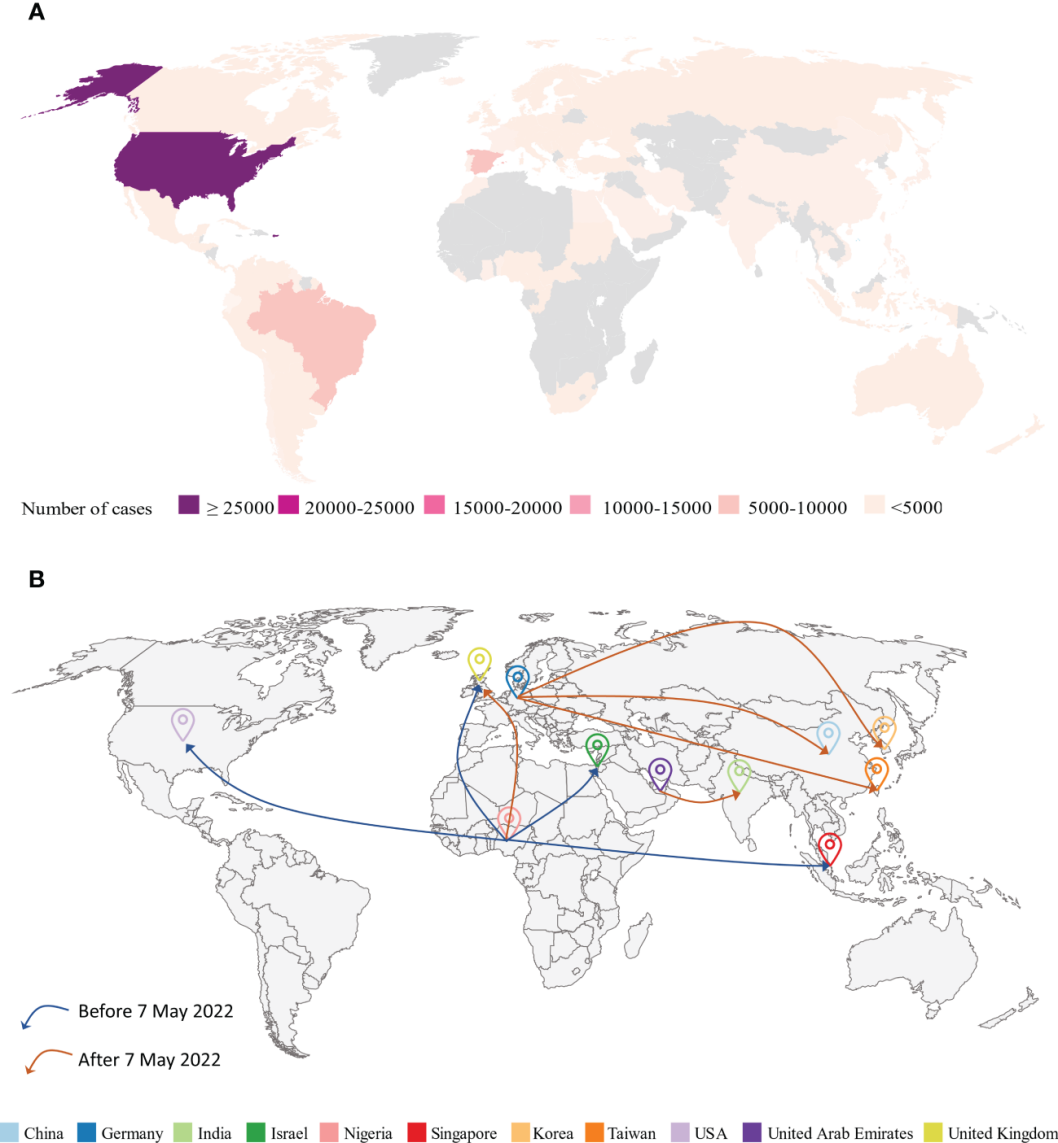
**(A)** Geographical distribution of confirmed and suspected monkeypox cases until February 20^th^, 2023 (Atlanta CDC). **(B)** Monkeypox virus (MPXV) airline transmission routes. Before May 7^th^, 2022, most of the travel-related cases in various countries (UK, Israel, Singapore, US) were from endemic areas like Nigeria. However, since May 7^th^, 2022, MPXV cases have dramatically increased worldwide and the majority of travel-related cases were from previous non-endemic areas such as Germany.

There has been a significant increase in MPXV cases worldwide since May 2022, particularly in Europe, the Americas, and Asia (**Figure 2A**). Owing to the rapid outbreak, the World Health Organization (WHO) has declared the MPXV outbreak a global health emergency (62). The Central African Republic has reported 5 clinical cases of secondary MPXV infection spread over 3 waves of intrafamilial infection (35). 9 cases of MPXV-associated deaths had been reported in Nigeria as of February 2023 (Atlanta CDC). No emergency measures, such as the release of smallpox vaccines from the global stockpile, have been adopted to control the further spread of the MPXV where it first emerged and spread (63). The concept of “One Health, One World” reminds us to monitor these endemic areas to prevent global pandemics (63). More than 30,000 confirmed cases and 30 MPXV-associated deaths have also been reported in the US through February 20^th^, 2023 (64). Of the 7 cases of MPXV infection reported in the UK from 2018 to 2021, 4 were imported *via* air travel, 1 was a healthcare worker, who caught the MPXV by caring for an infected patient, and the last 2 were household contacts (65). However, by 2022, the number of MPXV infections in the UK has dramatically increased. The UK had reported 3,735 cases by February 20^th^, 2023 (21). Currently, Chongqing, one of the China’s major cities, reported the first imported case of mpox in September 2022 (66). Additionally, MPXV has now been confirmed in South Korea and Japan (21). Current tends indicate that the MPXV is gradually spreading to Asia (67). The effects of the mpox epidemic on the population and society should not be underestimated or overlooked.

## 8 Impacts of global traveling

The global spread of MPXV can be attributed to community gatherings and frequent global travel across different continents. As global travel becomes more prevalent, human respiratory viruses such as SARS-CoV-2 (68), MERS-CoV (69, 70), and H7N9 (71) pose a threat to public health. For example, a single passenger infected with SARS-CoV-2 could cause a large cluster of cases during a long flight (72). Similarly, travel-related spread outside of Africa has caused global MPXV outbreaks (**Figure 2B**; **Table 3**).

**Table 3:** Cases of airline transmission.

Order of appearance	Monkeypox spread by air travel (name of the countries)		Date of spread	Remark	References
1	From Nigeria to the UK		October 2018	Two UK travelers reported a rash that started in the groin before leaving Nigeria.	(73)
2	From Nigeria to Israel		October 2018	After exposure to two dead rodents in Nigeria, the patient was infected.	(73)
3	From Nigeria to Singapore		May 7^th^, 2019	After the patient returned to Singapore from Nigeria, he was infected.	(74)
4	From Nigeria to the US		July 17^th^, 2021	After the patient returned to the USA from Nigeria, his skin samples tested positive for the West African clade of MPXV *via* PCR.	(75)
5	From Nigeria to Maryland, US		November 2021	After the patient returned to the US from Lagos, Nigeria, he was infected.	(76)
6	From Nigeria to the UK		May 7^th^, 2022	The patient developed a rash when arriving in the UK from Nigeria, MPXV was laboratory confirmed.	(24)
7	From Germany to Taiwan		June 16^th^, 2022	A 20-year-old returned to Taiwan from Germany. Then the CDC confirmed MPXV infection.	(77)
8	From Germany to Korean		June 21^st^, 2022	A 34-year-old Korean man traveled to Europe in June 2022. On the day returned to Korea, he presented with a genital lesion. Mpox was laboratory confirmed.	(78)
9	From United Arab Emirates (UAE) to India		July 2022	Case 1, developed a sore throat and worsening oral lesions after returning from URE to India. Case 2, his lesions spread to his face, back, neck, and forearm after traveling from UAE to Kerala, India.	(79)
10	From Germany to Chongqing, China		September 16^th^, 2022	A Chinese salesperson visited Germany and had MSM behavior, then returned to China and was confirmed with an MPXV infection.	(66)

### 8.1 The travel-related cases before the 2022 MPVX outbreak

In September 2017, Nigeria experienced a large and ongoing outbreak of MPXV (45), which may have caused MPXV transmission to previously non-endemic countries. Prior to the 2022 MPVX outbreak, most of the travel-related cases in various countries (UK, Israel, Singapore, and US) were from endemic areas such as Nigeria (73, 74, 76, 80). For instance, in October 2018, the UK reported one case of an infected individual who traveled from Nigeria to the UK with clinical signs of fever, lymphadenopathy, and a rash in the groin area the day before leaving Nigeria (81). This patient was detected MPXV-positive by multiple molecular assays and subsequently confirmed by sequencing (81). Similarly, one person, traveling from Nigeria to Israel, was reported with an infection after exposure to two dead rodents in Nigeria in October 2018 (73). Singapore reported one imported case of MPXV from Nigeria on May 7^th^, 2019 (74). On July 17^th^, 2021, the Texas reported one travel-related case of human MPXV from Nigeria (75). This was the first case of air travel-transmitted MPXV in the US (75).

### 8.2 The travel-related cases during the 2022 MPVX outbreak

Since May 7^th^, 2022, most travel-related cases have been reported from non-endemic areas such as Germany (66, 77, 78). These reports suggest that MPXV is still spreading in endemic areas. For example, MPXV cases have been identified and reported in Taiwan on June 20^th^, 2022; this case involved a young man who was studying in Germany and developed symptoms 4 days after arriving in Taiwan (77). Similarly, a passenger flying from Germany to Korea was identified as an MPXV-infected case on June 21^st^, 2022 (78). This was the first reported case of mpox in Korea (78). Similarly, Zhao et al. identified an individual infected with mpox in China, who had MSM behavior in Berlin and later returned to Chongqing (66).

## 9 Clinical implications and syndromes

**Figure 3:**
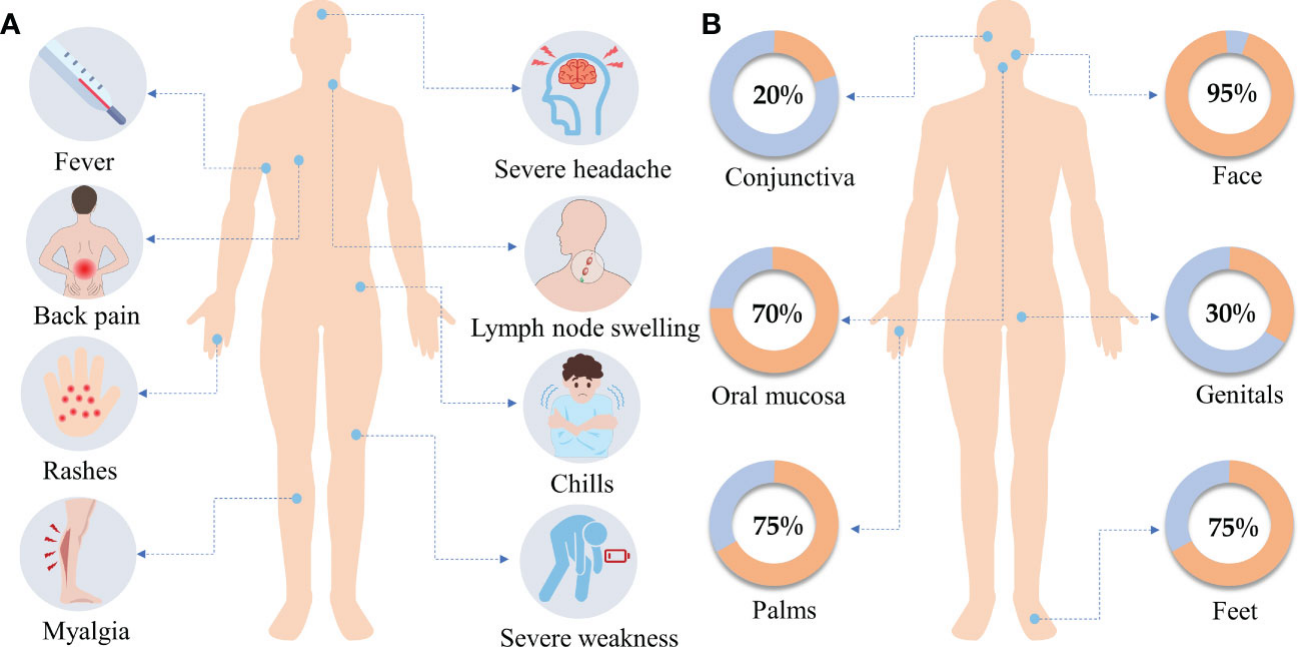
**(A)** Clinical symptoms. The main clinical symptoms of monkeypox include fever, severe headache, lymph node swelling, back pain, rashes, chills, myalgia, and severe weakness. **(B)** Rash distribution. Within 1 to 3 days of the onset of fever, the patient will develop skin lesions that affect the face (95%), the palms of the hands (75%), and the soles of the feet (75%), also affected are the oral mucosa (70%), the genitals (30%), the conjunctiva (20%). Data were obtained from WHO.

Clinical MPXV infections typically have two stages, the invasion phase (lasting 2−13 days) and the rash phase (lasting 7−24 days) (57). Symptoms may not appear for 6−10 days after MPXV infection (82). Mpox is a self-limiting disease with symptoms lasting between 2 and 4 weeks. The main symptoms during the invasion phase include fever (62%), severe headache (27%), lymphadenopathy (56%), and myalgia (31%) (82) (**Figure 3A**). Lymphadenopathy is the main characteristic of MPXV infection. Within 1 to 3 days of the onset of fever, the patient will develop skin lesions that affect the face (95%), palms (75%), feet (75%), oral mucosa (70%), genitals (30%), conjunctiva (20%), and cornea (43, 82) (**Figure 3B**). Notably, patients infected with MPXV in the 2022 outbreak occasionally developed symptoms that differed from typical mpox clinical manifestations, such as being diagnosed without fever or rash, with only one to a few skin lesions, or being characterized by anogenital lesions and rashes that spare the face and extremities (38, 46).

## 10 Methods to detect MPXV

The unprecedented MPXV outbreak emphasizes the importance of rapid and accurate diagnostics. The WHO recommends PCR for diagnosing MPXV during acute infection (82) (**Table 4**). The following points should be considered when using PCR in the laboratory to diagnose MPXV. First, skin lesion material, such as swabs of exudate or lesion surface, is the most ideal specimen type (89). Second, to avoid false positive testing results, CDC recommends repeated the testing to verify positive diagnostic results when a high Cycle Threshold (Ct) value (Ct ≥34) is obtained (90). Last, positive controls at low concentrations but above the limit of detection (LOD) should be included in the PCR assay of MPXV (89). In addition to PCR techniques, serum IgG and IgM assays are also feasible, however, these methods have certain limitations. The IgG detection, indicating previous exposure to the virus, may cause false positives owing to previous smallpox vaccination. The IgM detection, indicating recent exposure, may have caused serological cross-reaction with other orthopoxviruses. Individual orthopoxviruses cannot be distinguished using electron microscopy as they are morphologically similar and require large and expensive instruments. Additionally, mass spectrometry is expected to provide another method for detecting MPXV. Its rapid response, low analytical interference, better precision, and ease of multiplexing enable mass spectrometry to detect various pathogens and their variants (91).

**Table 4:** Laboratory PCR to diagnose monkeypox virus.

Testing methods	Gene targets	Targeted lineages	Primers/probes	Sequences	Characteristics	Ref
TaqMan-MGB (minor grove binding) real-time PCR	F3L	Monkeypox generic	F	5′-CTCATTGATTTTTCGCGGGATA-3′	Advantages: MPVX can be quickly and accurately distinguished from smallpox virus.	(83)
			R	5′-GACGATACTCCTCCTCGTTGGT-3′		
			P	5′-6FAM-CATCAGAATCTGTAGGCCGT-MGBNFQ-3′		
	N3R	Monkeypox generic	F	5′-AACAACCGTCCTACAATTAAACAACA-3′		
			R	5′-CGCTATCGAACCATTTTTGTAGTCT-3′		
			P	5′-6FAM-TATAACGGCGAAGAATATACT-MGBNFQ-3′		
Beacon based and MGB probe based real-time PCR	B6R	Monkeypox extracellular enveloped protein	F	5’-ATTGGTCATTATTTTTGTCACAGGAACA-3'	Limitation: the performance is related to the specific real-time PCR platform.	(84)
			R	5’-AATGGCGTTGACAATTATGGGTG-3'		
			P	5′-MGB/DarkQuencher-AGAGATTAGAAATA-FAM3′		
LightCycler quantitative PCR (LC-qPCR)	ATI	MPXV Congo clade specific	F	5′-GAGATTAGCAGACTCCAA-3'	Limitation: DNA sequencing after a PCR amplification is needed to differentiate MPXV West African and Congo Basin strains.	(85)
			R	5′-GATTCAATTTCCAGTTTGTAC-3'		
			P1	5′-GCAGTCGTTCAACTGTATTTCAAGATCTGAGAT-Fluorescein-3'		
			P2	5′-LCRed640-CTAGATTGTAATCTCTGTAGCATTTCCACGGC-Phos-3'		
	ATI	MPXV West African clade specific	F	5′-GAGATTAGCAGACTCCAA-3'		
			R	5′-TCTCTTTTCCATATCAGC-3'		
			P1	5′-GCAGTCGTTCAACTGTATTTCAAGATCTGAGAT-Fluorescein-3'		
			P2	5′-LCRed640-CTAGATTGTAATCTCTGTAGCATTTCCACGGC-Phos-3'		
TaqMan probe based Real‐time PCR	G2R	MPXV West African specific	F	5′-CACACCGTCTCTTCCACAGA-3'	Advantages: 1. Perform equally well in all platforms. 2. Capable of differentiating between Central and West African clades.	(86)
			R	5′-GATACAGGTTAATTTCCACATCG-3'		
			P	5′-FAM-AACCCGTCGTAACCAGCAATACATTT-BHQ1-3'		
	G2R	Monkeypox generic	F	5′-GGAAAATGTAAAGACAACGAATACAG-3'		
			R	5′-GCTATCACATAATCTGGAAGCGTA-3'		
			P	5′-FAM-AAGCCGTAATCTATGTTGTCTATCGTGTCC-BHQ1-3'		
	C3L	Monkeypox Congo basin-specific	F	5′-TGTCTACCTGGATACAGAAAGCAA-3'		
			R	5′-GGCATCTCCGTTTAATACATTGAT-3'		
			P	5′-FAM-CCCATATATGCTAAATGTACCGGTACCGGA-BHQ1-3'		
TaqMan probe based Real‐time PCR	F3L	Monkeypox generic	F	5′-CATCTATTATAGCATCAGCATCAGA-3'	Advantages: one-step, rapid and specific.	(87)
			R	5′-GATACTCCTCCTCGTTGGTCTAC-3'		
			P	5′-JOE-TGTAGGCCGTGTATCAGCATCCATT-BHQ1-3'		
Recombinase polymerase amplification (RPA) assay	G2R	Monkeypox generic	F	5′-AATAAACGGAAGAGATATAGCACCACATGCAC-3'	Advantages: Specificity: 100% Sensitivity: 95% Time: 3 to 10 minutes LOD: 16 molecules/μl	(88)
			R	5′-GTGAGATGTAAAGGTATCCGAACCACACG-3'		
			P	5′-ACAGAAGCCGTAATCTATGTTGTCTATCGQTFCCTCCGGGAACTTA-3'		

F, forward; R, reverse; P, probe.

## 11 Measures to prevent MPXV infection

The COVID-19 pandemic has taken a huge toll on the world economy and health, and we must monitor the spread of the MPXV to prevent the next pandemic (92). Bisanzioa et al. demonstrated that specific contact tracing and surveillance, isolation of confirmed cases, and ring vaccination substantially reduced the number of secondary cases by up to 86.1% while also shortening the duration of the MPXV outbreak by 75.7% (93).

### 11.1 Vaccines

Currently, several countries (including the UK, Canada, and the US) have decided to launch ring vaccination campaigns for high-risk individuals (94). Smallpox vaccination-induced antibodies can bind and recognize various orthopoxviridae proteins, providing cross-protection against MPXV (95). ACAM2000 is a live plaque-purified VACV derivative of Dryvax that has been FDA-approved for smallpox (96). JYNNEOS is a third-generation vaccine based on the non-replicating modified VACV Ankara (MVA) strain licensed by the FDA for the prevention of smallpox and mpox in adults (97). On May 24^th^, 2022, the US CDC decided to release JYNNEOS to control further outbreaks of MPXV. On August 9^th^, 2022, the US FDA issued an emergency use authorization of JYNNEOS in individuals younger than 18 years old at high risk of MPXV infection (98). In non-human primate experiments, ACAM2000 and JYNNEOS vaccines are effective in resisting the challenge of the MPXV (95). However, the efficacy of ACAM2000 and JYNNEOS vaccines in preventing MPXV resistance in humans needs to be further investigated.

### 11.2 Other prevention measures

Important measures should be taken to avoid any contact with infected animals and humans. It is essential to identify infected individuals and remind them to self-quarantine at home. When caring for patients with suspected or confirmed MPXV, healthcare personnel should wear protective clothing, gloves, eye protection, and N95 (or higher protection level) respirators (99). Similarly, airlines should take appropriate precautions to reduce the risk of infectious disease exposure for passengers, such as providing free hand sanitizer, and masks for passengers while flying (100). In addition, it is very important to maintain healthy sexual behavior, especially for MSM individuals.

It is also critical to raise public awareness about viral biology and MPVX transmission by providing basic information. Global efforts should also be encouraged and collaborated on to combat further MPXV transmission.

## 12 Perspective

Owing to the scarcity of smallpox vaccination and the population’s low immunity to orthopoxvirus, MPXV has the potential to become a widely transmitted human pathogenic virus, especially in the MSM community (101). Individual behaviors and public measures should be implemented for those vulnerable groups to prevent the massive spread of the virus at a community level. The species diversity and range of animal reservoir remain unknown. Further investigation is needed to identify the specific animal intermediate reservoirs of MPXV to prevent and control animal-to-human transmission. In addition, a well-controlled animal model is required to investigate whether the MPXV can be transmitted sexually. At a molecular level, it is necessary to explore how MPXV invades hosts and how cellular host immunity responds to MPXV. Furthermore, understanding unique receptors on host cells for MPVX would be critical and imperative for the development of antiviral drugs and preventive vaccines against the virus.

## 13 Search strategies and selection criteria

We searched PubMed, Google Scholar, WHO.int, and Web of Science for literature and case reports. A combination of the keywords “monkeypox virus”, “orthopoxvirus”, “genome”, “genetic evolution”, “transmission”, “reservoirs”, “detection”, “vaccines”, “prevention”, “syndromes”, “implication”, “vaccine”, “pregnant”, “vertical transmission”, “children”, “HIV”, “immune”, “newborns”, “MSM”, and “air travel” was used to retrieve related studies from 1985 to 2023, with over 80% references cited from the the last 2 years.
