# Corrigendum: Global transmission of monkeypox virus—a potential threat under the COVID-19 pandemic

**DOI:** 10.3389/fimmu.2025.1592351

**Published:** 2025-04-02

**Authors:** Yang Wang, Ping Leng, Hao Zhou

**Affiliations:** College of Medical Technology, Chengdu University of Traditional Chinese Medicine, Chengdu, China

**Keywords:** monkeypox virus, transmission, airline travel, detection, vaccination

In the published article, there was an error in **Figure 1** as published. We have misused the word “inappropriate” which can be perceived as inaccurate and non-scientific, and instead replaced it with the word “unprotected”. The corrected **Figure 1** and its caption appear below.

**Figure 1:**
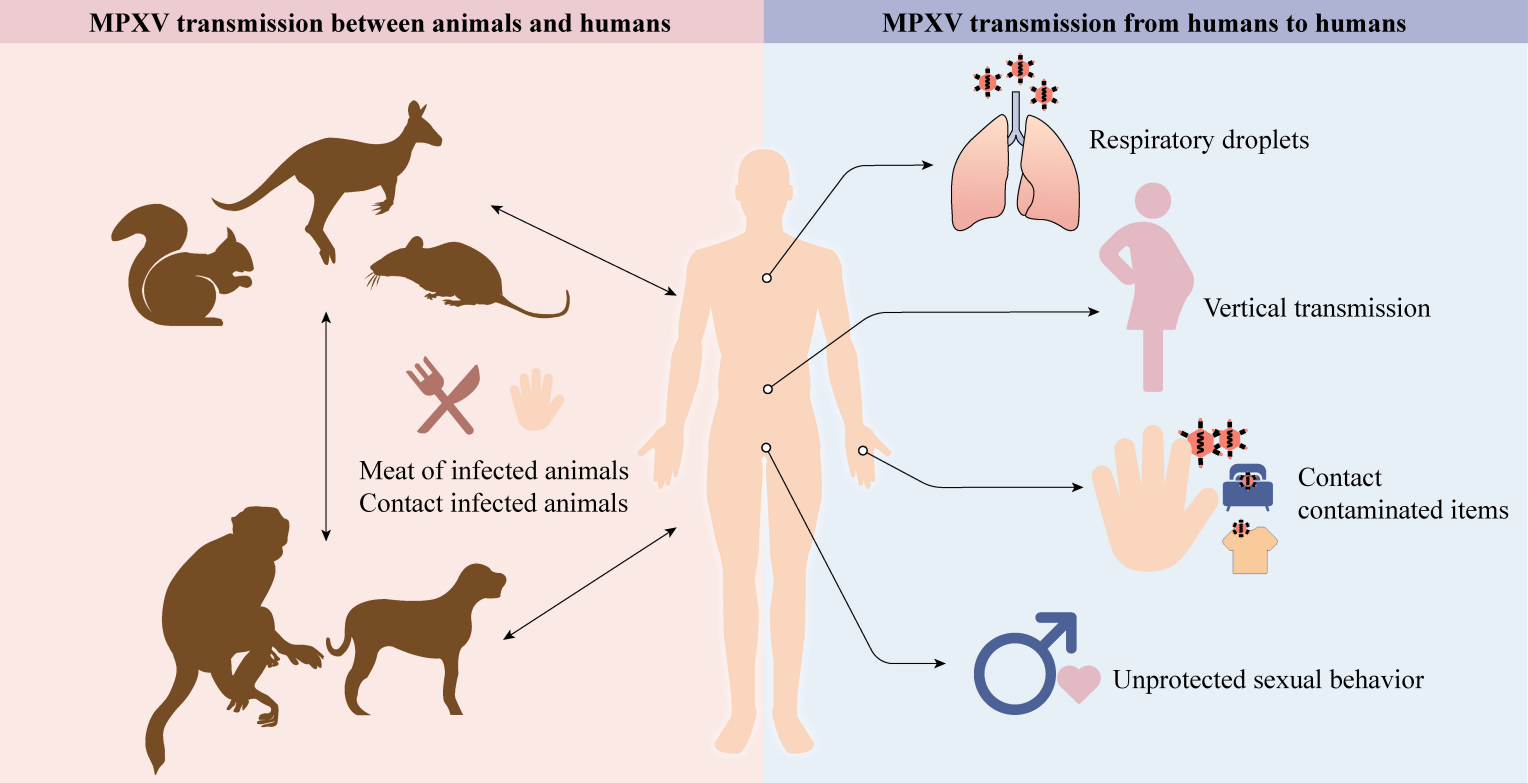
Animals, like rodents, dogs, and non-human primates, can spread MPXV to humans (cartoons modified from the SciDraw website). Respiratory droplets, vertical transmission, contact with contaminated items, and unprotected sexual behavior enable MPXV to transmit between humans.

In the published article, there was an error. We have misused the word “inappropriate”, which can be perceived as inaccurate and non-scientific. A correction has been made to **5.2 MPXV transmission from humans to humans**, *5.2.1 Sexual transmission*. This sentence previously stated:

“A recent meta-analysis including 124 MPXV cases demonstrated that inappropriate sexual behavior is the primary mode of transmission route (46).”

The corrected sentence appears below:

“A recent meta-analysis including 124 MPXV cases demonstrated that unprotected sexual behavior is the primary mode of transmission route (46).”

This sentence previously stated:

“For example, Antinori et al. reported that 4 infected young adults in Italy were exposed to inappropriate sexual encounters.”

The corrected sentence appears below:

“For example, Antinori et al. reported that 4 infected young adults in Italy were exposed to unprotected sexual encounters.”

The authors apologize for these errors and state that they do not change the scientific conclusions of the article in any way. The original article has been updated.

